# Cytokinin at the Crossroads of Abiotic Stress Signalling Pathways

**DOI:** 10.3390/ijms19082450

**Published:** 2018-08-19

**Authors:** Jaroslav Pavlů, Jan Novák, Vladěna Koukalová, Markéta Luklová, Břetislav Brzobohatý, Martin Černý

**Affiliations:** 1 Department of Molecular Biology and Radiobiology, Faculty of AgriSciences, Mendel University in Brno, 613 00 Brno, Czech Republic; pavlu@mendelu.cz (J.P.); jan.novak@mendelu.cz (J.N.); vladena19@seznam.cz (V.K.); luklovam@gmail.com (M.L.); brzoboha@ibp.cz (B.B.); 2 CEITEC—Central European Institute of Technology, Faculty of AgriSciences, Mendel University in Brno, 613 00 Brno, Czech Republic; 3 Institute of Biophysics AS CR, 612 00 Brno, Czech Republic; 4 Phytophthora Research Centre, Faculty of AgriSciences, Mendel University in Brno, 613 00 Brno, Czech Republic

**Keywords:** cytokinin, abiotic stress, temperature, drought, nutrient, stress tolerance

## Abstract

Cytokinin is a multifaceted plant hormone that plays major roles not only in diverse plant growth and development processes, but also stress responses. We summarize knowledge of the roles of its metabolism, transport, and signalling in responses to changes in levels of both macronutrients (nitrogen, phosphorus, potassium, sulphur) and micronutrients (boron, iron, silicon, selenium). We comment on cytokinin’s effects on plants’ xenobiotic resistance, and its interactions with light, temperature, drought, and salinity signals. Further, we have compiled a list of abiotic stress-related genes and demonstrate that their expression patterns overlap with those of cytokinin metabolism and signalling genes.

## 1. Introduction

**Figure 1:**
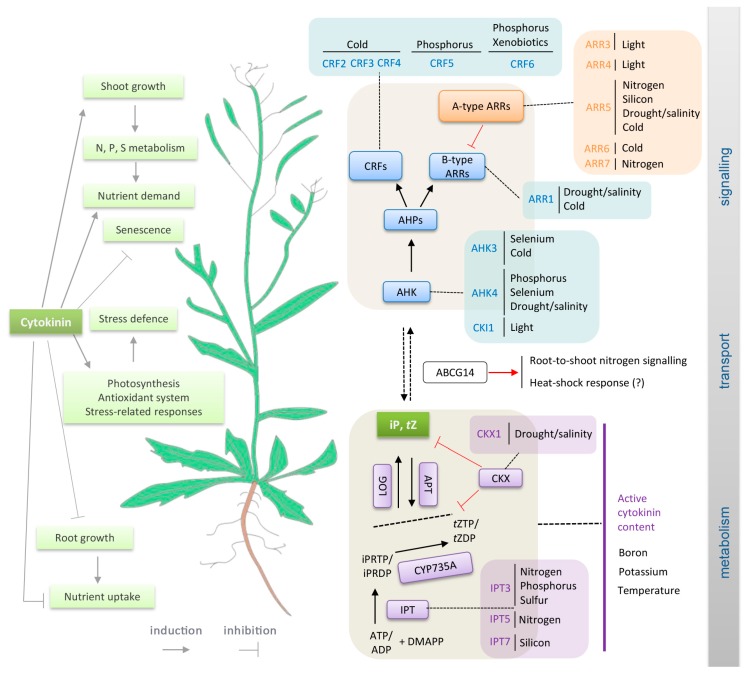
Crosstalk between abiotic stress signals and cytokinin. Summary of interactive points between cytokinin metabolism and signalling pathways (as currently modelled in *Arabidopsis* [5]) and abiotic stress response pathways. See corresponding sections of the text for details and references.

As sessile organisms, plants have evolved elaborate mechanisms that enable them to sense and respond to changes in environmental conditions, and are thus crucial for their adaptation and survival. These mechanisms involve abiotic stimuli triggering wide arrays of local and long-distance signals initiating developmental processes and stress responses that are regulated and coordinated by common integrative pathways. One of the key transmitted signals is cytokinin: a multifaceted plant hormone that plays major roles in diverse plant growth and development processes. Cytokinin signalling cascades are evolutionarily related to the two-component systems in unicellular organisms that participate in transduction of signals that are triggered by various environmental stimuli, for example, changes in temperature, nutrient levels, chemoattractants, or osmotic conditions [1,2,3]. In contrast to ethylene, another phytohormone that is involved in a two-component signalling pathway, cytokinin was not traditionally considered part of the primary stress response machinery. However, more recently, cytokinin crosstalk with ethylene and other so-called “stress hormones” (jasmonate, salicylic acid and abscisic acid) has been recognized (e.g., [4]), and current evidence indicates that it could be a primary perceptor in temperature or nutrient sensing. In the following text, we present an overview of cytokinin crosstalk with abiotic stimuli, as outlined in Figure 1. The presented evidence includes findings from compilation of a list of abiotic stress-related genes and analyses showing that their expression patterns overlap with those of cytokinin metabolism and signalling genes. Similarities in expression profiles mentioned here are expressed as percentages that are derived by multivariate analysis from average profiles.

## 2. Nutrient Stress

Plants require a number of elements for their growth and development. Besides carbon, hydrogen, and oxygen, which are primarily obtained from carbon dioxide and water, plants actively take up at least 20 elements. These include both macronutrients (nitrogen, phosphorus, sulphur, potassium), and micronutrients (including boron, iron, silicon and selenium). As discussed in the following text, cytokinin plays a pivotal role in plants’ uptake of these nutrients, and their responses to toxic metal(loid)s, including cadmium, aluminium, and arsenite.

### 2.1. Nitrogen

**Figure 2:**
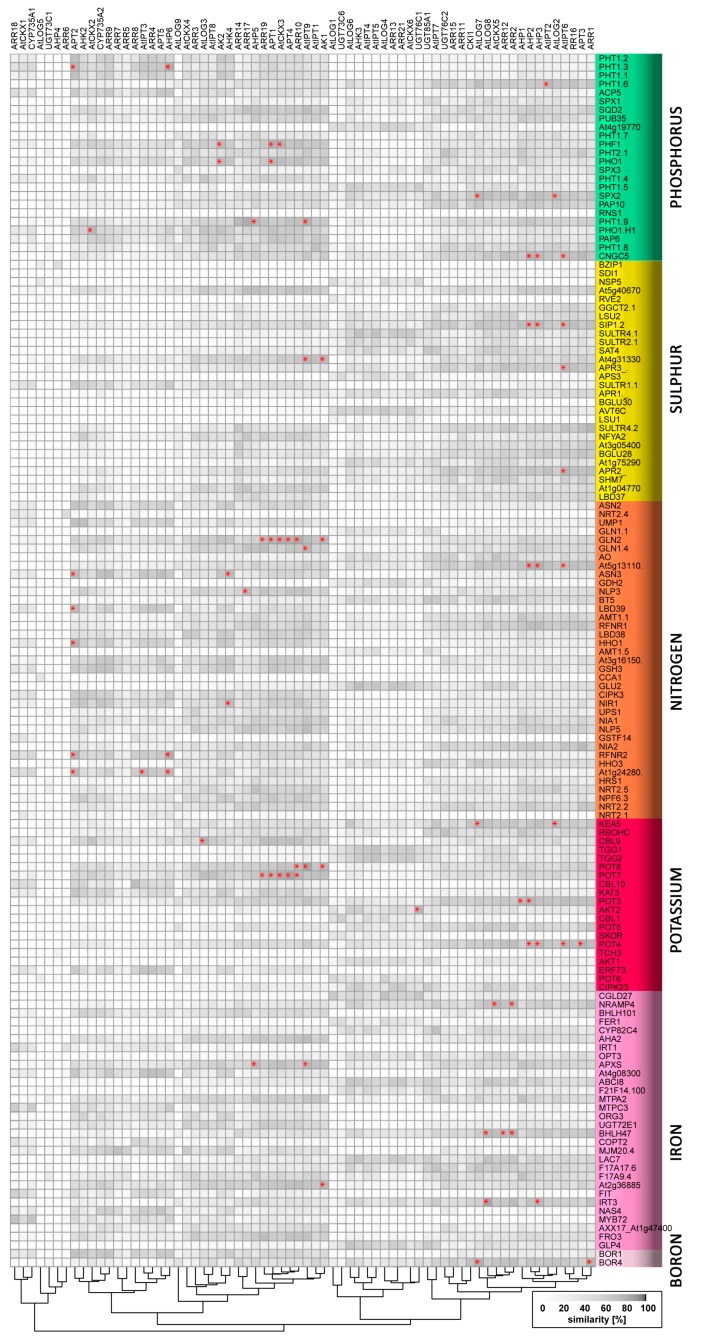
Similarity of expression patterns of genes related to nutrient stress and genes involved in cytokinin metabolism or signalling. Asterisks indicate profile similarities >85%. The heatmap was generated using R software and available data from Araport [33].

**Figure 3:**
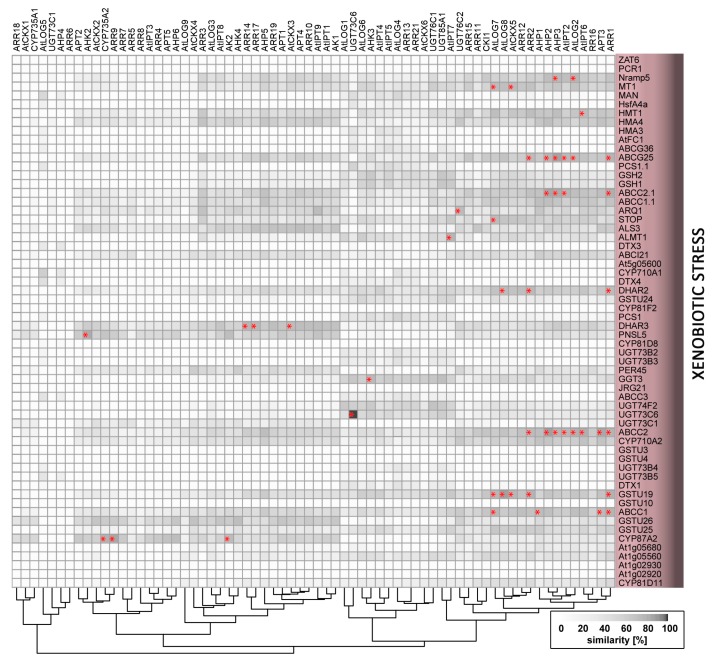
Similarity of expression patterns of genes related to xenobiotic stress and genes involved in cytokinin metabolism or signalling. Asterisks indicate profile similarities >85%. The heatmap was generated using R software and available data from Araport [33].

**Figure 4:**
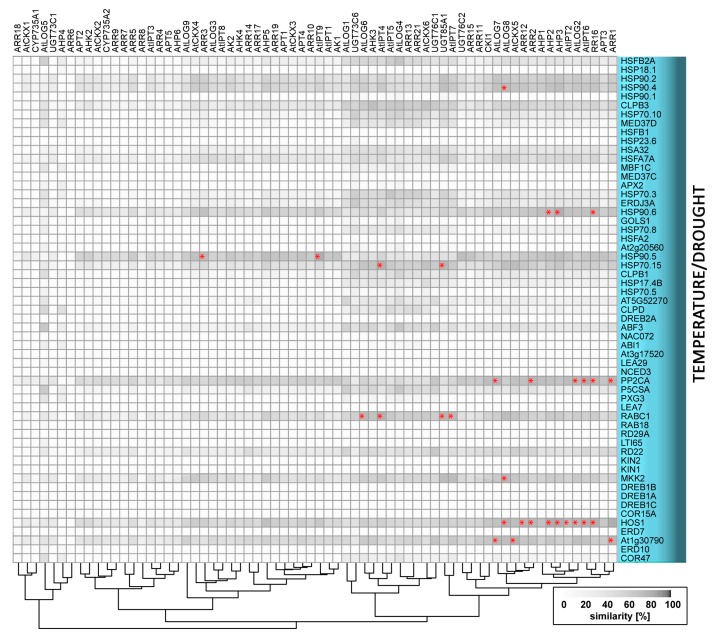
Similarity of expression patterns of genes related to temperature/drought stress and genes involved in cytokinin metabolism or signalling. Asterisks indicate profile similarities >85%. The heatmap was generated using R software and available data from Araport [33].

Nitrogen is one of the most strongly growth-limiting nutrients for plants. Thus, their internal nitrogen status and both the availability and distribution of nitrogen in their growth media are sensed by a complex network of signalling pathways that generate and regulate integrated responses to local and long-distance signals, including several phytohormones [6,7,8,9]. A well-known connection between nitrogen metabolism and cytokinin is nitrate supplementation-induced cytokinin biosynthesis in the roots. In *Arabidopsis*, the availability of nitrate regulates cytokinin biosynthesis rates by controlling the expression of the enzymes that catalyse the first rate-limiting step, isopentenyl transferase (IPT3, IPT5), and subsequent production of *trans*-Zeatin (*t*Z)-type cytokinins, cytochrome P450 (CYP735A2) [10,11]. In addition to these cytokinin metabolism genes, genes encoding cytokinin-responsive type-A response regulators (*ARR*s) and Cytokinin Response Factors (*CRF*s) are regulated by nitrate, but not ammonium, in *Arabidopsis* [12,13,14,15]. The signalling components that are involved in nitrate-upregulated cytokinin biosynthesis are the nitrate transporter-receptor NRT1 (*NPF6.3*) acting upstream of *IPT3* [16], and the *NLP-NIGT1* transcriptional cascade controlling *CYP735A2* and *IPT3* expression [17]. Cytokinin also participates in nitrate foraging, which involves plants’ preferential development of lateral roots in nitrate-rich areas, thereby maximizing nitrate acquisition [18,19,20]. The transcription factor TCP20, which controls the nitrate foraging response [9,21], can also bind to promoters of type-A *ARR5/7*, providing an additional link between nitrogen and cytokinin signalling [18,19]. Thus, the disruption of cytokinin signalling affects nitrate uptake, as demonstrated in the *Arabidopsis* cytokinin signalling mutant *arr1,10,12*. In this genotype, the nitrate-mediated induction of glutaredoxin genes (*GRX*) responsible for nitrate-mediated induction of primary root growth is abolished [22]. Moreover, RNA silencing of *AtGRX3/4/5/7/8* has demonstrated that GRXs act downstream of cytokinin in a signal transduction pathway, which, in this case, suppresses plants’ primary root growth when nitrate supplies are sufficient [22,23]. Our meta-analysis (which included a comparison of expression patterns of all known cytokinin metabolism and signalling genes to those of 43 genes that respond to nitrogen deficiency) provided further evidence of cytokinin’s involvement in nitrate signalling. Expression patterns of seven and eight cytokinin signalling and metabolism genes, respectively, showed high similarity (>85%) to those of nitrogen deficiency genes. Overlaps were the strongest for the cytokinin biosynthetic gene *APT2*, which had a similar expression profile to five nitrogen-deficiency genes. For details, see Table 1 and Figure 2, Figure 3 and Figure 4.

In the shoot, root-derived cytokinins have been shown to mediate nitrate responses and modulate key traits, such as leaf size [24,25] and meristem activity-related traits [26]. Following its nitrate-induced synthesis in the root, cytokinin acts as a long-distance (systemic) signal, conveying information about the root’s nitrogen status that influences shoot metabolism and growth [12,27,28,29,30]. Cytokinin translocation via xylem in this systemic nitrogen response system is mediated by the cytokinin transporter ABCG14, and recent transcriptomic analysis indicates that its target could be the glutamate/glutamine metabolism machinery in the shoot [20,31]. Recent findings also show that long-distance transport of the cytokinin precursor *t*ZR (which has low activity) can account for nitrate availability-mediated adjustments of shoot apical meristem size and organogenesis rates through modulating the expression of WUSCHEL [32]. Root-to-shoot cytokinin signalling operates in both directions, and, for example, lateral root growth is regulated by *t*Z content in the shoot [18,19,20].

### 2.2. Phosphorus

Like nitrogen sensing, a complex signalling system is required to maintain inorganic phosphate (Pi) homeostasis, and plants’ responses to Pi-limiting conditions involve multiple phytohormones [34,35]. As in nitrate sensing, one of the strongly affected cytokinin genes is the biosynthetic gene *IPT3*. Pi shortage causes the downregulation of *IPT3* [36] and cytokinin signalling components, including the cytokinin receptor *AHK4* [37]. Conversely, the resupply of Pi after a shortage causes upregulation of *IPT3*, *CRF5,* and *CRF6* [38]. Moreover, reductions in root cytokinin levels upregulate the expression of Pi transporters [39,40,41,42] and the exogenous supply of cytokinin can suppress Pi uptake and Pi starvation responses in *Arabidopsis* and rice [37,43,44,45,46,47,48], presumably by mobilizing Pi from internal sources (preferentially stores in shoot tissues) [46]. This may temporarily reduce Pi starvation signalling and contribute to the relief of Pi deficiency symptoms, including the reported moderation of shoot growth inhibition of Pi-starved plants in the presence of cytokinin [49]. It has been proposed that the level of cell-cycle activity governs the magnitude of Pi demand in Pi-starved plants. This would fit well with cytokinin’s opposite effects on cell cycling in shoot and root meristems, where it, respectively, stimulates and represses cell division [50]. Further, auxin-cytokinin crosstalk via the auxin responsive factor OsARF16 regulates Pi signalling, and transport of Pi from roots to shoots [47].

### 2.3. Potassium

Potassium is the most abundant inorganic cation in plants, and it is one of the primary macronutrients that are generally added (together with nitrogen and phosphorus) to soil in fertilizers. Analysis of *Arabidopsis* plants has shown that potassium deprivation reduces cytokinin contents, and cytokinin signalling regulates root growth inhibition and potassium uptake [51]. The cited authors also found that cytokinin-deficient plants have enhanced the tolerance of potassium deficiency, which they attributed to the stimulation of ROS accumulation, root hair growth, and expression of *HAK5*, which encodes a potassium uptake transporter. This transporter connects multiple phytohormonal networks, as it is also regulated by ethylene [52] and participates in the modulation of the auxin transporter PIN1’s localization [53]. We found no significant similarity between expression patterns of *HAK5* and any cytokinin metabolism/signalling genes. However, expression patterns of genes encoding seven potassium-deficiency-related genes (four potassium transporters, two antiporters, and a potassium channel) showed ≥85% similarity to those of candidate cytokinin genes (Table 1, Figure 2).

### 2.4. Sulphur

The availability of sulphur in soil is directly associated with crop yields and quality, and sulphur deficiency induces a number of adaptive responses [54]. A link between sulphur deficiency and responses in cytokinin status is indicated by *IPT3* downregulation in roots of *Arabidopsis* plants grown on sulphur-deficient media [36], and observed changes in cytokinin contents triggered by sulphur deficiency in poplar [55]. In addition, exogenous application of cytokinin upregulates expression of sulphur-responsive genes in leaves [36]. By contrast, cytokinin downregulates the root expression of sulphate transporters (*SULTR1;1* and *SULTR1;2*) that are involved in sulphate acquisition from the soil [39,56], and the *arr1,10,12* triple mutant displays sulphur-deficiency-like gene expression patterns [57]. Thus, complex interplay between cytokinin and sulphur signalling, which is possibly mediated by independent regulatory circuits, is likely involved. The sulphur-deficiency marker gene *GGCT2;1* encodes a key enzyme of glutathione degradation and it is a highly cytokinin-responsive gene [58], suggesting that cytokinin may participate in glutathione homeostasis and cytokinin-mediated glutathione decomposition may play a physiologically important role in nutrient mobilization.

### 2.5. Boron

Boron is an essential micronutrient for the growth of higher plants but there is a very narrow range between deficient and toxic concentrations [59]. Symptoms of severe boron deficiency include root growth inhibition, perturbances in root morphology, and reductions in vegetative and reproductive growth. Early detectable changes in boron-deficient plants include disturbances of hormonal metabolism and several lines of evidence suggest that ethylene and auxin are involved in the regulation of boron stress responses [60]. Boron deprivation induces the downregulation of cytokinin signalling genes [61,62], and our meta-analysis showed that *BOR4*, encoding a boron transporter, has a similar expression pattern to *ARR1* and the cytokinin metabolism gene *LOG7*. Moreover, in oilseed rape, the shoot boron concentration reportedly correlates closely with cytokinin content, and boron enhances both cytokinin synthesis and the conversion of weakly active cytokinins to highly active forms [63]. Conversely, recent analysis indicates that boron deficiency inhibits root meristem growth via a molecular mechanism involving the cytokinin-mediated repression of cyclin CYCD3 [64].

### 2.6. Iron

Cytokinin suppresses expression of several genes that respond to iron deficiency in *Arabidopsis* [65]. This cytokinin-induced repression is mediated via AHK3 and AHK4 receptors, and it targets genes encoding components of the iron-uptake machinery (*FRO2*, *IRT1*) and the iron-deficiency induced transcription factor FIT1. The repression does not reflect the plant’s iron nutritional status, and analysis of a *fit1* loss-of-function mutant indicates that it acts via a distinct, FIT1-independent signalling pathway [65]. This could be mediated by the ARF16 transcription factor, which is required for iron deficiency responses in rice [66] and participates in the auxin-cytokinin control of phosphate homeostasis [47]. Only five of 38 selected genes that are related to iron-deficiency had similar expression profiles to cytokinin regulatory genes. Moreover, the expression pattern of *NRAMP4* (encoding a transporter of iron and several other metals) is similar to that of *CKX* (encoding a cytokinin degradation enzyme) and *ARR1*, but *NRAMP4* expression is not reportedly upregulated by exogenous application of cytokinin [65]. Thus, this coregulation is unlikely to reflect iron status signalling.

### 2.7. Silicon

Silica minerals are major soil components, and high silicon uptake, boosted by root silicon transporters, promotes plants’ tolerance to many biotic and abiotic stresses. Mineralized (insoluble) silica provides structural support for many plants, but it can also enhance various defence mechanisms of plants and influence their stress responses by modulating their hormonal balance [67,68]. The beneficial effects of silicon are partially mediated by cytokinin [69]. *Inter alia*, silicic acid induces the cytokinin synthesis gene *IPT7* and silicon accumulation delays dark-induced leaf senescence through the activation of cytokinin pathways in sorghum and *Arabidopsis* [70].

### 2.8. Selenium

At low concentrations, selenium promotes plant growth and stress resistance [71,72], but elevated levels can be toxic. Selenate and selenite, the two major forms of selenium that are found in the environment, are readily absorbed by plants via sulphate and phosphate transporters, respectively [73]. In this respect, cytokinin-regulated sulphate and Pi pathways might form a point of cross-talk between selenium and cytokinin signalling. For instance, the cytokinin-responsive sulphate transporter SULTR1;2 is a determinant of selenium tolerance in *Arabidopsis* [74]. Cytokinin signalling is promoted in the root tip of selenite-exposed *Arabidopsis* plants, and high cytokinin levels reportedly improve the performance of selenite-exposed roots, whereas reductions in cytokinin status or sensitivity enhance selenite sensitivity [75,76,77]. Recently, a selenium-tolerant *Arabidopsis* mutant with a loss-of-function mutation in a terpenoid synthase gene (TPS22) has been described. Observed effects of the mutation include reductions in cytokinin levels and the expression of cytokinin receptors *AHK3* and *AHK4*, while the application of exogenous cytokinin upregulated selenocysteine methyltransferase (as well as high-affinity phosphate transporters) and decreased selenium tolerance of the mutant [42].

### 2.9. Xenobiotics

Strict control of processes that are involved in plants’ absorption, translocation, and storage of essential metals is crucial for the maintenance of their concentrations within physiological ranges and the avoidance of toxicity. Nevertheless, despite the transport systems’ selectivity, they may also take up toxic, non-essential metals and metalloids, such as arsenic, cadmium, chromium, lead, and mercury. Responses to these toxic xenobiotics, including cadmium [78] and aluminium [79], involve increases in cytokinin biosynthesis and signalling that inhibit root growth. Accordingly, application of substances that reduce active cytokinin contents or signalling can mitigate the adverse effects of cadmium [80]. Similarly, the cytokinin signalling component CRF6 is induced by organic xenobiotics, including the herbicide atrazine [81,82] and atrazine inhibition is weaker in the *crf6* insertional mutant line than in wild-type plants [83]. Moreover, cytokinin-deficient plants grown in cadmium-contaminated soil reportedly accumulate more cadmium [39] and display enhanced arsenate tolerance [41], which is likely due to higher levels of thiol compounds [41]. Cytokinin also induces the upregulation of glutathione-*S*-transferase GSTU26 [84,85] and may thus play a role in glutathione conjugation.

## 3. Cytokinin Roles in Drought and Salinity Tolerance

Drought and salinity stress are the most frequent abiotic stresses and both impair crop production on a global scale [86]. Analysis of natural variants of *Arabidopsis* has shown that even mild drought can adversely affect plants if they are not evolutionarily adapted to it [87]. Plants react to water-limiting conditions by reducing their cytokinin levels, mainly through the modulation of cytokinin metabolism—as shown (*inter alia*) in *Arabidopsis*, creeping bentgrass, soybean, tobacco, and sunflower [88,89,90,91,92,93]—and/or the regulation of cytokinin receptors’ expression [94,95]. However, other mechanisms, including activation of the negative regulators of cytokinin signalling AHP6 and ARR5 also probably participate in this process [94,96,97]. Appropriate modulation of cytokinin metabolism and signalling has been known to improve drought and salt tolerance for many years [92,95,98,99], and at least five mechanisms may contribute to cytokinin-mediated enhancement of tolerance of water deficiency. These are: protection of the photosynthetic machinery, enhancement of antioxidant systems, improvement in water balance regulation, modulation of plant growth and differentiation, and modulation of activities of stress-related phytohormones.

### 3.1. Cytokinin Modulates Photosynthesis under Water-Limiting Conditions and Salt Stress

Changes in cytokinin status (mainly increases in cytokinin levels) reportedly enhance photosynthesis and related processes under water-deficiency or salt stress in many plant species [99,100,101,102,103,104], by increasing the expression of genes that are involved in photosynthesis, chlorophyll levels, photochemical efficiency, photochemical quenching, electron transport rates, and/or CO_2_ assimilation. Accordingly, in transgenic barley plants ectopically expressing the cytokinin-degradation enzyme AtCKX1, reductions in CO_2_ assimilation rates, accompanied by lower stomatal conductance, have been recorded [105]. Conversely, increases in CO_2_ assimilation have been observed in barley lines overexpressing *CKX* under a different promoter resulting in localization to different compartments. However, the cited authors only presented results from plants with elevated concentrations of *t*Z-type cytokinins [106]. It has been previously demonstrated that *CKX* overexpression stimulates cytokinin biosynthesis [107], so the observed positive effect on CO_2_ assimilation was likely due to increases in cytokinin content.

### 3.2. Cytokinin Enhances Capacities of Antioxidant Systems

Ectopic expression of *ipt* reportedly increases the capacities of plants’ antioxidant systems, including levels of antioxidants during severe drought stress [100]. This could protect their cells from excessive stress-induced ROS accumulation, thereby preserving chloroplast integrity [100,108,109] and reducing electrolyte leakage and/or rises in malondialdehyde levels [57,89,110]. On the other hand, ectopic expression of *CKX* in barley has been found to activate genes putatively involved in flavonoid biosynthesis [105] and flavonoids also participate in drought tolerance [111]. These effects of cytokinin in drought stress tolerance could involve indirect priming of antioxidant systems in response to manipulation of cytokinin homeostasis. In accordance with this hypothesis, significant enhancement of cytokinin biosynthesis can induce hypersensitivity-like responses and ROS-mediated cell death [112].

### 3.3. Cytokinin Influences Water Balance Regulation

Clearly, water management is crucial for drought tolerance, and plants with low levels of cytokinin or weak cytokinin signalling generally have higher water contents during drought stress than counterparts with higher cytokinin contents or stronger signalling [92,95,105]. This could be due to better root systems, since cytokinin is a known negative regulator of root growth and lateral root formation [106,110]. The improved water uptake in these plants is clearly complemented with reductions in transpiration rates and stomatal apertures, which could protect them from severe water losses during stress periods [57,105,110]. Ectopic expression of *ipt* also reduces water losses in plants that are exposed to drought, even when they have higher transpiration rates and stomatal conductance, but the mechanisms that are involved are elusive [90,102].

### 3.4. Cytokinin Effects on Growth

As cytokinins play key roles in root and shoot development they also participate in expression of growth and architectural traits that are required for tolerance of water-limiting conditions [113]. Cytokinins are well known to reduce root to shoot hypocotyl ratios [39,114,115], and one of the approaches for enhancing plants’ drought tolerance is to decrease cytokinin levels in order to modify root morphology and enhance root biomass [116]. Root-specific overexpression of *CKX* can also enhance root growth, nutrient uptake, and drought tolerance [106], as well as improving recovery after drought stress [116] without adverse effects on shoot growth. Similarly, one of the dehydration-responsive element binding factors in *Malus* (MdDREB6.2) activates the expression of *MdCKX*, mainly in roots, and overexpression of this factor can enhance drought tolerance [110]. Several studies indicate that not only quantitative features but also qualitative traits of root tissues could be important factors in cytokinin-regulated responses to water-limiting conditions, including the differentiation of vascular tissue [117] and lignification [116].

### 3.5. Cytokinin Crosstalk with Stress-Related Phytohormones

#### 3.5.1. Abscisic Acid

Rapid accumulation of the phytohormone abscisic acid plays a crucial role in regulating plants’ defensive responses to drought stress, including stomatal closure, growth modulation, and synthesis of protective metabolites. It has been known for more than a decade that cytokinin and abscisic acid have antagonistic functions in diverse physiological processes, including stress tolerance, germination, and hypocotyl greening [95,118,119]. Cytokinin signalling has been shown to be dramatically inhibited by abscisic acid application [120,121] and cytokinin facilitates degradation of the abscisic acid signalling component transcription factor ABI5 [118]. It has been reported that under drought stress plants with decreased levels of cytokinin or attenuated cytokinin signalling have decreased levels of abscisic acid, but higher sensitivity to this stress-related hormone and greater drought tolerance [57,92,122]. However, elucidation of the molecular mechanism involved in this interaction has begun only recently. Experiments with a series of cytokinin and abscisic acid signalling mutants have demonstrated that cytokinin and abscisic acid interact directly through their signalling components, as plants constitutively expressing *HA-Flag-ARR5* and *arr5* loss-of-function mutants respectively showed increased and attenuated sensitivity to abscisic acid treatment [97]. ARR5 stability is promoted by phosphorylation catalysed by SnRK2 protein kinases that are key components of the abscisic acid signalling pathway. In contrast, type-B ARR1, 11, and 12 interact with these SnRK2s and repress their kinase activity, and the abscisic acid hypersensitivity of the triple mutant *arr1,11,12* can be completely rescued by mutation of SnRK2s [97]. Interestingly, the same authors found that expression of ARR1ΔDDK (a constitutively activated form of ARR1), but not constitutive expression of *ARR1-Myc*, was associated with slight insensitivity to abscisic acid, suggesting that the modulation of ARR1’s phosphorylation status by cytokinin signalling may also be important. Since cytokinin is essential for normal growth of plants [10] the SnRK2-ARR regulatory module is clearly a recently discovered signalling hub that balances growth and defence in response to environmental cues.

#### 3.5.2. Jasmonates

Jasmonic acid is known to play a role in drought tolerance [123,124]. *Inter alia*, drought-induced xylem differentiation is negatively and positively regulated by cytokinin and jasmonic acid, respectively, and jasmonic acid attenuates cytokinin signalling by repressing the cytokinin receptor *AHK4* and stimulating expression of *AHP6*, a negative regulator of cytokinin signalling [117]. In addition, cytokinin may influence jasmonate metabolism. Ectopic expression of *AtCKX1* in barley plants has been found to induce expression of lipoxygenases, which participate in the release of volatile compounds, including jasmonates [105], but an increase in jasmonic acid has also been observed in tobacco plants with highly increased levels of cytokinin [112].

## 4. Temperature and Cytokinin

Temperature is one of the most important abiotic factors influencing plants’ growth, development, productivity, and yields. Plants can only grow within taxa-specific temperature ranges, thus suboptimal temperatures cause stress, and temperature limits their geographical distributions. The mechanisms that are involved in temperature perception and signalling in plants are far from completely understood, but key aspects of associated morphological changes are clearly mediated by phytohormones.

### 4.1. Low Temperature Stress: Cold and Freezing

Most reported responses of cytokinin metabolism or signalling systems to low temperatures in plants are repressive [94,125,126,127,128], but there are some documented exceptions, notably cold-mediated upregulation of *AHK3* [95]. However, the responses are complex, and roles of cytokinin and cytokinin signalling pathways in cold tolerance are unclear. Cold-induced attenuation of cytokinin signalling seems to impair plants’ tolerance of low temperature because the exogenous application of cytokinin significantly promotes cold tolerance in *Arabidopsis* [129,130,131]. Accordingly, recent hormonal analysis of *Zoysia* grass has shown that a genotype from relatively high latitude retained higher cytokinin levels during low-temperature treatment and exhibited higher freezing tolerance than a genotype from a lower latitude [128]. However, this seems to conflict with a reported negative role of AHK cytokinin receptors in cold tolerance [129]. The mechanisms whereby cytokinin could both promote cold tolerance and activate negative regulators of cold stress responses is unclear, but it seems to be at least partially independent of the cold-induced CBF/DREBs regulatory system [129].

Cold has also been shown to transiently activate expression of type-A *ARRs* in a cytokinin- and ethylene-dependent manner [129,130]. Mutation of *ARR5*, *ARR6*, and *ARR7* leads to higher freezing tolerance [129], but the overexpression of *ARR7* reportedly has both negative [129] and positive effects [130]. Results of overexpression studies indicate that other type-A ARRs [130] and ARR22, a cold-inducible type-C ARR [131], may also play positive roles in freezing tolerance. As shown by these conflicting results, the molecular mechanisms involved are unclear and further research is required. Besides *ARRs,* cytokinin response factors (CRFs) that act downstream of the primary cytokinin signalling pathway participate in responses to low temperature. More specifically, CRFs are induced by cold in *Arabidopsis* and tomato [130,132], and detailed analysis of *Arabidopsis* overexpressors and mutants has shown that CRF4 mediates freezing tolerance in non-acclimated plants [132], while CRF2 and CRF3 regulate lateral root development in response to cold stress [133]. Our meta-analysis revealed two novel candidates for interactive points in cytokinin-cold stress crosstalk: the MAP kinase MKK2, and the component of the ubiquitin-proteasome pathway, HOS1. The gene encoding HOS1 has a similar expression pattern to four and five genes that are involved in cytokinin metabolism and signalling, respectively (Figure 4). Cytokinin and its signalling evidently play important roles in cold stress responses, but various aspects of the molecular mechanism of their action regarding (for example) the duration of the period of cytokinin modulation prior to the stress require clarification

### 4.2. High Temperature and Heat Stress

Observed responses of *Arabidopsis* to heat stress treatments include a rapid but transient increase in active cytokinin contents [134,135]. A rapid proteomic heat-shock response that could be mimicked to some extent by cytokinin treatment at standard temperature has also been reported [136], indicating that cytokinin may play a role in temperature perception. Moreover, the accumulation of cytokinin has been observed in *Pinus radiata* under prolonged heat stress and in recovered plants [137,138]. Plants with increased levels of cytokinins show a higher accumulation of heat-shock proteins [107,139,140] and enhanced activity of the antioxidant system [88,112]. Accordingly, transgenic lines with inactivated components of cytokinin signalling pathways or reductions in pools of active cytokinin have displayed increased tolerance to high temperatures [141,142]. Further, analyses of temperature-induced hypocotyl growth in cytokinin-deficient transgenic plants and cytokinin receptor *ahk* double mutants have shown that impairment of the cytokinin pathway strongly inhibits growth at high temperatures [136]. This indicates that cytokinin could serve as a signal for thermomorphogenesis. It is also likely that a higher temperature sensitizes cytokinin signalling, which could explain why a transient increase in the active cytokinin pool is followed by its significant depletion [134,135], and downregulation in cytokinin metabolism genes and the expression of ARR-type A orthologs in strawberry (*Fragaria vesca*) [143,144]. It has also been proposed that heat stress-induced cytokinin depletion can promote stomatal closure, as this process is inhibited in plants with increased cytokinin levels [135].

## 5. Light Signalling and the Circadian Clock Interact with Cytokinin

**Figure 5:**
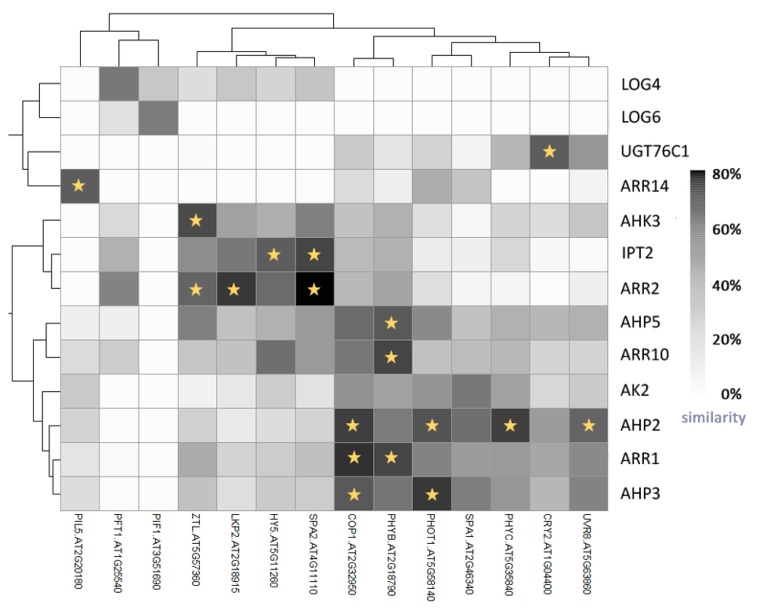
Heatmap showing degrees of similarity between expression profiles of cytokinin (signalling and metabolism) genes and genes involved in light perception. The heatmap was generated using R software and data available from Araport [33]. Asterisks indicate >70% similarity.

As described in a recent review [145], soon after its discovery it was found that cytokinin promotes chlorophyll synthesis and chloroplast development. There is increasing evidence of direct interactions between cytokinin and light via the light photoreceptor phyB [146,147]. Moreover, cytokinin-mediated development in *Arabidopsis* is modulated by the expression of the sensor histidine kinase CKI1 (Cytokinin Independent-1), which is regulated by phyA (and thus light) via the phyA interacting factor (PIF3) and Circadian Clock Associated 1 (CCA1) [148]. Further, levels of the cytokinin pool in tobacco leaves vary diurnally, with the main peak occurring around midday [149], and a key component of the circadian clock in plants, Late Elongated Hypocotyl (LHY), modulates cytokinin levels in *Populus* trees [150]. Reductions in cytokinin status or sensitivity enhance circadian stress in *Arabidopsis* and cytokinin-deficient plants display a highly similar expression of clock output genes to that of clock mutants [151]. Light conditions may also influence contents of specific cytokinins, as recently demonstrated in detached leaf experiments [152]. Moreover, light-cytokinin interactions are not limited to cytokinin metabolism and components of the two-component cytokinin responsive pathway. They also influence a bZIP transcription factor, Elongated Hypocotyl 5 (HY5), which participates in photomorphogenesis [94,153]. Cytokinin is also apparently involved in photoprotection mechanisms as plants with deficiencies in cytokinin receptors or cytokinin signalling are more susceptible to light stress than wild-type counterparts [154,155]. To identify new candidate participants in light-cytokinin interactions, we subjected *Arabidopsis* transcriptomic expression profiles to association analysis. Data were collected from the publicly available database Thalemine (available online: http://apps.araport.org/thalemine/), then normalized, and degrees of similarity between expression patterns were visualized in a heatmap (Figure 5). In total, we tested similarities in expression profiles of 70 candidate cytokinin genes and 31 genes that were putatively involved in light signalling. The analysis revealed that 10 of the latter had similar expression patterns (>70%) to candidate cytokinin genes. The results confirmed the previously described relation between *ARR* and *COP1*, but also highlighted several novel putative interactions, including a connection between the UV-B receptor UVR8 and the AHP2 component of cytokinin signalling. This is consistent with a recent finding that cytokinin regulates UV-B-induced damage in tomato seedlings [156].

## 6. Summary

Generally, it can be concluded that cytokinin metabolism and signalling play important roles in abiotic stress tolerance and the manipulation of these processes in crops could be beneficial for sustainable agriculture. However, recent studies have mainly focused on global transcriptomic, proteomic and metabolomic changes in various plant species with modulated cytokinin levels [105,122,250]. Thus, further detailed analysis is required to confirm the importance of identified candidate genes/proteins and validate their roles in stress tolerance. Moreover, current models have substantial gaps. There is mounting evidence of intensive crosstalk in phytohormonal signalling, including redox and proteasome-ubiquitin pathways [251]. Thus, any disruption in a single phytohormone signalling pathway will probably affect the whole hormonome, but current limits in the sensitivity and spatiotemporal scope of analyses constrain our ability to detect all of the changes. Ongoing advances in hormonome analyses will undoubtedly improve our understanding [252], but another limitation is that most presented findings are based solely on transcriptomic analyses, in some cases supplemented with results of knocking out or overexpressing specific genes. Furthermore, posttranslational modifications play important roles in regulatory networks [253], and thus abiotic responses [254]. Thus, they must also be considered. Similarly, to fully understand phytohormonal interactions in abiotic stress responses, it will be crucial to integrate protein-protein interactions and the associated signalling hubs and networks [255].

## Figures and Tables

**Table 1:** List of all abiotic stress-related genes, with references, and their putative interactions with cytokinin according to the expression profile analysis outlined in Figure 2, Figure 3 and Figure 4. Genes in bold indicate profile similarities >85% to cytokinin-related genes, and numbers indicate the number of detected co-expressed cytokinin signalling/metabolism genes.

Gene Name	AGI Code	UniProt Protein Name	Significant Co-Expression with Cytokinin Signalling/Metabolism Genes	References
NITROGEN				
*NPF6.3*	AT1G12110	Protein NRT1/PTR FAMILY 6.3		[157]
*NRT2.1*	AT1G08090	High-affinity nitrate transporter 2.1		[158]
*NRT2.2*	AT1G08100	High-affinity nitrate transporter 2.2		[157]
*NRT2.4*	AT5G60770	High affinity nitrate transporter 2.4		[159,160]
*NRT2.5*	AT1G12940	High affinity nitrate transporter 2.5		[13,157,161]
*AMT1-1*	AT4G13510	Ammonium transporter 1 member 1		[157]
*AMT1-5*	AT3G24290	Putative ammonium transporter 1 member 5		[161,162]
*GDH2*	AT5G07440	Glutamate dehydrogenase 2		[157]
*GSH3*	AT3G03910	Probable glutamate dehydrogenase 3		[157]
***GLN2***	**AT5G35630**	**Glutamine synthetase**	2/4	[157,158]
*GLU2*	AT2G41220	Ferredoxin-dependent glutamate synthase 2		[163]
*NIA1*	AT1G77760	Nitrate reductase [NADH] 1		[13,158]
*NIA2*	AT1G37130	Nitrate reductase [NADH] 2		[157]
***NIR1***	**AT2G15620**	**Ferredoxin–nitrite reductase**	1/0	[13,158]
*UMP1*	AT5G40850	Urophorphyrin methylase 1		[13,157,158]
*GLN1-1*	AT5G37600	Glutamine synthetase cytosolic isozyme 1-1		[159,164]
***GLN1-4***	**AT5G16570**	**Glutamine synthetase cytosolic isozyme 1-4**	0/1	[157,164]
*At3g16150*	AT3G16150	Probable isoaspartyl peptidase/l-asparaginase 2		[157,159]
*ASN2*	AT5G65010	Asparagine synthetase [glutamine-hydrolyzing] 2		[22,157]
***ASN3***	**AT5G10240**	**Asparagine synthetase [glutamine-hydrolyzing] 3**	1/1	[165]
***At5g13110***	**AT5G13110**	**Glucose-6-phosphate 1-dehydrogenase 2**	2/1	[157,158]
***At1g24280***	**AT1G24280**	**Glucose-6-phosphate 1-dehydrogenase 3**	1/2	[22,158]
*UPS1*	AT2G03590	Ureide permease 1		[159]
*AT4G39795*	AT4G39795	Uncharacterized protein		[159]
*RFNR1*	AT4G05390	Ferredoxin–NADP reductase		[14,158]
***RFNR2***	**AT1G30510**	**Ferredoxin–NADP reductase**	1/1	[158]
*GSTF14*	AT1G49860	Glutathione S-transferase F14		[158]
*BT5*	AT4G37610	BTB/POZ and TAZ domain-containing protein 5		[158]
*CCA1*	AT2G46830	Protein CCA1		[157]
*TGA1*	AT5G65210	Transcription factor TGA1		[166]
*TGA4*	AT5G10030	Transcription factor TGA4		[166]
***NLP3***	**AT4G38340**	**Protein NLP3**	1/0	[167]
*NLP5*	AT1G76350	Protein NLP5		[167]
*NLP7*	AT4G24020	Protein NLP7		[167]
***HHO1***	**AT3G25790**	**Transcription factor HHO1**	0/1	[158,160]
*HRS1*	AT1G13300	Transcription factor HRS1		[158,160]
*HHO3*	AT1G25550	Transcription factor HHO3		[22,158,160]
*LBD37*	AT5G67420	LOB domain-containing protein 37		[14,158,168]
*LBD38*	AT3G49940	LOB domain-containing protein 38		[14,158,168]
***LBD39***	**AT4G37540**	**LOB domain-containing protein 39**	0/1	[158,168]
*CIPK3*	AT2G26980	CBL-interacting serine/threonine-protein kinase 3		[158]
*CIPK13*	AT2G34180	CBL-interacting serine/threonine-protein kinase 13		[157]
*AO*	AT5G14760	l-aspartate oxidase		[158]
PHOSPHORUS				
***PHO1***	**AT3G23430**	**Phosphate transporter PHO1**	0/2	[38]
***PHO1-H1***	**AT1G68740**	**Phosphate transporter PHO1 homolog 1**	0/1	[169]
***PHF1***	**AT3G52190**	**SEC12-like protein 1**	0/3	[169]
*PHT1-1*	AT5G43350	Inorganic phosphate transporter 1-1		[170,171]
*PHT1-2*	AT5G43370	Probable inorganic phosphate transporter 1-2		[171,172]
***PHT1-3***	**AT5G43360**	**Probable inorganic phosphate transporter 1-3**	1/1	[38,171]
***PHT1-4***	**AT2G38940**	**Inorganic phosphate transporter 1-4**	1/0	[171,172]
***PHT1-5***	**AT2G32830**	**Probable inorganic phosphate transporter 1-5**	1/0	[170,171]
***PHT1-6***	**AT5G43340**	**Probable inorganic phosphate transporter 1-6**	0/1	[172]
*PHT1-7*	AT3G54700	Probable inorganic phosphate transporter 1-7		[173]
*PHT1-8*	AT1G20860	Probable inorganic phosphate transporter 1-8		[172,173]
***PHT1-9***	**AT1G76430**	**Probable inorganic phosphate transporter 1-9**	1/1	[172,173]
*PHT2-1*	AT3G26570	Inorganic phosphate transporter 2-1		[170,172]
*SPX1*	AT5G20150	SPX domain-containing protein 1		[169,174]
***SPX2***	**AT2G26660**	**SPX domain-containing protein 2**	0/2	[174]
*SPX3*	AT2G45130	SPX domain-containing protein 3		[38]
*IPS1*	AT3G09922	INDUCED BY PHOSPHATE STARVATION1		[169,174]
*F12E4_330*	AT5G03545	At5g03545		[169]
*ACP5*	AT5G27200	Acyl carrier protein 5		[38]
*RNS1*	AT2G02990	Ribonuclease 1		[169,170]
*SQD2*	AT5G01220	Sulfoquinovosyl transferase SQD2		[169]
*PAP10*	AT2G16430	Purple acid phosphatase 10		[38]
*PAP6*	AT1G56360	Purple acid phosphatase 6		[38]
*At4g19770*	AT4G19770	Glycosyl hydrolase family protein with chitinase insertion domain-containing protein		[38]
*PUB35*	AT4G25160	U-box domain-containing protein 35		[38]
*GDPD3*	AT5G43300	Glycerophosphodiester phosphodiesterase GDPD3		[38]
*ETC3*	AT4G01060	MYB-like transcription factor ETC3		[38]
SULPHUR				
*SULTR1;1*	AT4G08620	Sulfate transporter 1.1		[175,176]
*SULTR2;1*	AT5G10180	Sulfate transporter 2.1		[175,176]
*SULTR4;1*	AT5G13550	Sulfate transporter 4.1		[177,178]
*SULTR4;2*	AT3G12520	Probable sulfate transporter 4.2		[178,179,180,181]
*APS3*	AT4G14680	ATP-sulfurylase 3		[176,178]
*APR1*	AT4G04610	5′-adenylylsulfate reductase 1		[176,178]
***APR2***	**AT1G62180**	**5′-adenylylsulfate reductase 2**	0/1	[178]
***APR3***	**AT4G21990**	**5′-adenylylsulfate reductase 3**	0/1	[178,180]
*SAT4*	AT4G35640	Serine acetyltransferase 4		[181,182]
*BGLU28*	AT2G44460	Beta-glucosidase 28		[179,180]
*BGLU30*	AT3G60140	Beta-glucosidase 30		[180,182]
*SDI1*	AT5G48850	Protein SULFUR DEFICIENCY-INDUCED 1		[178,179]
*SDI2*	AT1G04770	Protein SULFUR DEFICIENCY-INDUCED 2		[176,178]
*SHM7*	AT1G36370	Serine hydroxymethyltransferase 7		[178,180]
*GGCT2;1*	AT5G26220	Gamma-glutamylcyclotransferase 2-1		[178,180,182]
*LSU1*	AT3G49580	Protein RESPONSE TO LOW SULFUR 1		[178,180]
*LSU2*	AT5G24660	Protein RESPONSE TO LOW SULFUR 2		[178,180,182]
*At3g05400*	AT3G05400	Sugar transporter ERD6-like 12		[180,182]
***At4g31330***	**AT4G31330**	**Protein of unknown function**	0/2	[180,182]
***SIP1-2***	**AT5G18290**	**Probable aquaporin SIP1-2**	2/1	[180]
*At5g40670*	AT5G40670	Cystinosin homolog		[180]
*At1g75290*	AT1G75290	NAD		[180]
*NSP5*	AT5G48180	Nitrile-specifier protein 5		[180]
*AVT6C*	AT3G56200	Amino acid transporter AVT6C		[178,180]
*NFYA2*	AT3G05690	Nuclear transcription factor Y subunit A-2		[180]
*BZIP1*	AT5G49450	Basic leucine zipper 1		[180]
*RVE2*	AT5G37260	Homeodomain-like superfamily protein		[180]
POTASSIUM				
*POT5*	AT4G13420	Potassium transporter 5		[182,183]
***POT4***	**AT3G02050**	**Potassium transporter 4**	2/2	[183]
*AKT1*	AT2G26650	Potassium channel AKT1		[184,185]
*RBOHC*	AT5G51060	Respiratory burst oxidase homolog protein C		[183]
*CIPK23*	AT1G30270	CBL-interacting serine/threonine-protein kinase 23		[183,186]
*TGG1*	AT5G26000	Myrosinase 1		[182]
*TGG2*	AT5G25980	Myrosinase 2		[182]
*POT6*	AT1G70300	Potassium transporter 6		[187]
***POT8***	**AT5G14880**	**Potassium transporter 8**	1/2	[184,187]
***KEA5***	**AT5G51710**	**K^+^ efflux antiporter 5**	0/2	[183]
*KAT3*	AT4G32650	Potassium channel KAT3		[184]
*SKOR*	AT3G02850	Potassium channel SKOR		[184,188,189]
***AKT2***	**AT4G22200**	**Potassium channel AKT2/3**	0/1	[189]
***POT3***	**AT4G23640**	**Potassium transporter 3**	2/0	[190]
***POT7***	**AT5G09400**	**Potassium transporter 7**	2/3	[191]
*CBL1*	AT4G17615	Calcineurin B-like protein 1		[189]
***CBL9***	**AT5G47100**	**Calcineurin B-like protein 9**	0/1	[189]
*CBL10*	AT4G33000	Calcineurin B-like protein 10		[183]
*TCH3*	AT2G41100	Calcium-binding EF hand family protein		[192]
*ERF73*	AT1G72360	Integrase-type DNA-binding superfamily protein		[183]
IRON				
*AXX17_At1g47400*	AT1G47400	Uncharacterized protein		[182,193]
*At1g47395*	AT1G47395	At1g47390		[182]
*AT2G14247*	AT2G14247	Expressed protein		[182]
*At1g13609*	AT1G13609	Defensin-like		[182]
*IRT1*	AT4G19690	Fe^2+^ transport protein 1		[182,193,194]
*F17A17.6*	AT3G07720	AT3g07720/F17A17_6		[182,193,194]
*MTPA2*	AT3G58810	Metal tolerance protein A2		[193,194]
*MJM20.4*	AT3G12900	2-oxoglutarate		[193,194]
*F21F14.100*	AT3G61930	Uncharacterized protein At3g61930/F21F14_100		[193,194]
*COPT2*	AT3G46900	Copper transporter 2		[193,194]
*CYP82C4*	AT4G31940	Cytochrome P450 82C4		[193]
*GLP4*	AT1G09560	Germin-like protein subfamily 2 member 1		[193,194]
*F17A9.4*	AT3G06890	At3g06890		[193,194]
*UGT72E1*	AT3G50740	UDP-glycosyltransferase 72E1		[193,194]
*ORG3*	AT3G56980	Transcription factor ORG3		[182,193,194]
*MYB72*	AT1G56160	Transcription factor MYB72		[193,194]
*MTPC3*	AT3G58060	Putative metal tolerance protein C3		[193,194]
*FIT*	AT2G28160	Transcription factor FER-LIKE IRON DEFICIENCY-INDUCED TRANSCRIPTION FACTOR		[194,195]
***BHLH47***	**AT3G47640**	**Transcription factor bHLH47**	2/1	[193,195]
*BHLH101*	AT5G04150	Transcription factor bHLH101		[182,193]
*NAS4*	AT1G56430	Probable nicotianamine synthase 4		[182,193]
*OPT3*	AT4G16370	Oligopeptide transporter 3		[195,196]
*CGLD27*	AT5G67370	Protein CONSERVED IN THE GREEN LINEAGE AND DIATOMS 27		[182]
*FRO2*	AT1G01580	Ferric reduction oxidase 2		[182]
*FRO3*	AT1G23020	Ferric reduction oxidase 3		[193,195]
*AHA2*	AT4G30190	Plasma membrane ATPase		[195]
***NRAMP4***	**AT5G67330**	**Metal transporter Nramp4**	1/1	[193,195]
*FER1*	AT5G01600	Ferritin-1		[193,194]
*ABCI8*	AT4G04770	UPF0051 protein ABCI8		[193,194]
***At2g36885***	**AT2G36885**	**Translation initiation factor**	0/1	[193,194,195]
***APXS***	**AT4G08390**	**Stromal ascorbate peroxidase**	1/1	[193,194]
*LAC7*	AT3G09220	Laccase-7		[193,194,195]
***IRT3***	**AT1G60960**	**Fe^2+^ transport protein 3, chloroplastic**	1/1	[193]
*At4g08300*	AT4G08300	WAT1-related protein At4g08300		[182]
*FRO4*	AT5G23980	Ferric reduction oxidase 4		[185]
BORON				
*BOR1*	AT2G47160	Boron transporter 1		[197]
***BOR4***	**AT1G15460**	**Boron transporter 4**	1/1	[198]
TEMPERATURE/DROUGHT				
*RD29A*	AT5G52310	Low-temperature-induced 78 kDa protein		[141,199,200]
*KIN1*	AT5G15960	Stress-induced protein KIN1		[201,202]
*KIN2*	AT5G15970	Stress-induced protein KIN2		[203,204]
*COR15A*	AT2G42540	Protein COLD-REGULATED 15A		[200,201,202]
*COR47*	AT1G20440	Dehydrin COR47		[201,202]
*ERD10*	AT1G20450	Dehydrin ERD10		[201,202]
*ERD7*	AT2G17840	Protein EARLY-RESPONSIVE TO DEHYDRATION 7		[201,202,205]
***At1g30790***	**AT1G30790**	**F-box protein At1g30790**	0/2	[205]
*MKK2*	AT4G29810	Mitogen-activated protein kinase kinase 2		[206]
*RAB18*	AT5G66400	Dehydrin Rab18		[207,208,209]
*LTI65/RD29B*	AT5G52300	Low-temperature-induced 65 kDa protein		[129,199,200]
*RD22*	AT5G25610	BURP domain protein RD22		[207,209]
***HOS1***	**AT2G39810**	**E3 ubiquitin-protein ligase HOS1**	4/3	[203,210]
*DREB1B*	AT4G25490	Dehydration-responsive element-binding protein 1B		[201,202]
*DREB1C*	AT4G25470	Dehydration-responsive element-binding protein 1C		[201,202,209]
*DREB1A*	AT4G25480	Dehydration-responsive element-binding protein 1A		[201,202]
***RABC1***	**AT1G43890**	**Ras-related protein RABC1**	0/2	[202]
*CLPD*	AT5G51070	Chaperone protein ClpD		[209,211]
*SWEET15*	AT5G13170	Bidirectional sugar transporter SWEET15		[40,209]
*P5CSA*	AT2G39800	Delta-1-pyrroline-5-carboxylate synthase A		[40,209]
*ABI1*	AT4G26080	Protein phosphatase 2C 56		[209]
*DREB2A*	AT5G05410	Dehydration-responsive element-binding protein 2A		[209,212]
*NCED3*	AT3G14440	9-cis-epoxycarotenoid dioxygenase NCED3		[200,209]
*ABF3*	AT4G34000	ABSCISIC ACID-INSENSITIVE 5-like protein 6		[209,213]
***PP2CA***	**AT3G11410**	**Protein phosphatase 2C 37**	2/2	[200,209,214]
*PXG3/RD20*	AT2G33380	Probable peroxygenase 3		[200,209,211]
*LEA7*	AT1G52690	Late embryogenesis abundant protein 7		[209,215]
*LEA29*	AT3G15670	Late embryogenesis abundant protein 29		[40]
*At3g17520*	AT3G17520	Late embryogenesis abundant protein		[208,209]
*NAC072*	AT4G27410	NAC domain-containing protein 72		[208,209]
*MBF1C*	AT3G24500	Multiprotein-bridging factor 1c		[216,217]
*HSFA2*	AT2G26150	Heat stress transcription factor A-2		[217,218,219]
*HSA32*	AT4G21320	Protein HEAT-STRESS-ASSOCIATED 32		[216,217,218]
*CLPB1*	AT1G74310	Chaperone protein ClpB1		[216,217,218]
*CLPB3*	AT5G15450	Chaperone protein ClpB3		[216,218]
*HSFB2A*	AT5G62020	Heat stress transcription factor B-2a		[216,219]
*HSFA7A*	AT3G51910	Heat stress transcription factor A-7a		[217,218,219]
*HSP90-1*	AT5G52640	Heat shock protein 90-1		[216,217,218]
*HSP90-2*	AT5G56030	Heat shock protein 90-2		[216,217]
*At2g20560*	AT2G20560	At2g20560/T13C7.15		[216,217,218]
*HSFB1*	AT4G36990	Heat stress transcription factor B-1		[216,217,218]
*HSP23.6*	AT4G25200	23.6 kDa heat shock protein		[217,218]
*HSP18.1*	AT5G59720	18.1 kDa class I heat shock protein		[217,218]
*HSP17.4B*	AT1G54050	17.4 kDa class III heat shock protein		[216,217]
*MED37C*	AT3G12580	Probable mediator of RNA polymerase II transcription subunit 37c		[217,220]
*HSP70-5*	AT1G16030	Heat shock 70 kDa protein 5		[217,218,220]
*HSP70-10*	AT5G09590	Heat shock 70 kDa protein 10		[216,217]
*GOLS1*	AT2G47180	Galactinol synthase 1		[216,217,218]
*APX2*	AT3G09640	L-ascorbate peroxidase 2		[217,218]
*ERDJ3A*	AT3G08970	DnaJ protein ERDJ3A		[216,217]
***HSP90-6***	**AT3G07770**	**Heat shock protein 90-6**	2/0	[216,217]
*HSP90-4*	AT5G56000	Heat shock protein 90-4		[216,217]
*HSP70-8*	AT2G32120	Heat shock 70 kDa protein 8		[216,217]
*MED37D*	AT5G02490	Probable mediator of RNA polymerase II transcription subunit 37c		[216,217]
*HSP70-3*	AT3G09440	Heat shock 70 kDa protein 3		[216,217]
***HSP70-15***	**AT1G79920**	**Heat shock 70 kDa protein 15**	0/2	[216,217]
***HSP90-5***	**AT2G04030**	**Heat shock protein 90-5**	0/1	[216,217]
**XENOBIOTIC STRESS**				
*GSH1*	AT4G23100	Glutamate–cysteine ligase		[221,222]
*GSH2*	AT5G27380	Glutathione synthetase		[221,222]
*PCS1*	AT5G44070	Glutathione gamma-glutamylcysteinyltransferase 1		[221,222]
*MAN3*	AT3G10890	Mannan endo-1		[223]
*ZAT6*	AT5G04340	Zinc finger protein ZAT6		[223]
*PCR1*	AT1G14880	Protein PLANT CADMIUM RESISTANCE 1		[224]
*HMA3*	AT4G30120	Putative inactive cadmium/zinc-transporting ATPase HMA3		[225,226]
***HMA4***	**AT2G19110**	**Putative cadmium/zinc-transporting ATPase HMA4**	2/1	[227]
*HSFA4A*	AT4G18880	Heat stress transcription factor A-4a		[228]
*FC1*	AT5G26030	Ferrochelatase-1		[222]
***HMT-1***	**AT3G25900**	**Homocysteine S-methyltransferase 1**	2/2	[229]
***MT1A***	**AT1G07600**	**Metallothionein-like protein 1A**	1/4	[230]
***NRAMP5***	**AT4G18790**	**Metal transporter Nramp5**	2/2	[226,231]
*ABCG36*	AT1G59870	ABC transporter G family member 36		[232]
***ABCB25***	**AT5G58270**	**ABC transporter B family member 25**	4/3	[233]
***ABCC1***	**AT1G30400**	**ABC transporter C family member 1**	4/2	[226,234,235]
***ABCC2***	**AT2G34660**	**ABC transporter C family member 2**	10/7	[226,234,235]
***HAC1***	**AT2G21045**	**Protein HIGH ARSENIC CONTENT 1**	1/1	[236]
*ALMT1*	AT1G08430	Aluminum-activated malate transporter 1		[237,238]
***ALS3***	**AT2G37330**	**Protein ALUMINUM SENSITIVE 3**	2/4	[237,239]
***STOP1***	**AT1G34370**	**Protein SENSITIVE TO PROTON RHIZOTOXICITY 1**	0/1	[237,239]
*CYP81D11*	AT3G28740	Cytochrome P450 81D11		[240,241,242,243]
*CYP710A1*	AT2G34500	Cytochrome P450 710A1		[82,244,245]
*CYP81D8*	AT4G37370	Cytochrome P450		[81,243,244,245]
*UGT73B2*	AT4G34135	UDP-glucosyl transferase 73B2		[241,242,244]
*UGT73B3*	AT4G34131	UDP-glycosyltransferase 73B3		[242,244]
*UGT73B4*	AT2G15490	UDP-glycosyltransferase 73B4		[241,242,244]
*UGT73C1*	AT2G36750	UDP-glycosyltransferase 73C1		[241]
*GSTU3*	AT2G29470	Glutathione S-transferase U3		[241,244]
*GSTU10*	AT1G74590	Glutathione S-transferase U10		[82,243,246]
***GSTU19***	**AT1G78380**	**Glutathione S-transferase U19**	2/3	[243,247]
*GSTU24*	AT1G17170	Glutathione S-transferase U24		[240,241,242,243]
*GSTU25*	AT1G17180	Glutathione S-transferase U25		[241,242,243,244]
*GSTU26*	AT1G17190	Glutathione S-transferase U26		[248]
***GGT4***	**AT4G29210**	**Glutathione hydrolase 3**	1/0	[249]
*ABCC3*	AT3G13080	ABC transporter C family member 3		[244]
*ABCI21*	AT5G44110	ABC transporter I family member 21		[242,243]
*DTX1*	AT2G04040	Protein DETOXIFICATION 1		[243,245]
*DTX3*	AT2G04050	Protein DETOXIFICATION 3		[243,245]
*DTX4*	AT2G04070	Protein DETOXIFICATION 4		[241,245]
*CYP710A2*	AT2G34490	Cytochrome P450 710A2		[82]
***DHAR2***	**AT1G75270**	**Glutathione S-transferase DHAR2**	1/0	[243,244]
***DHAR3***	**AT5G16710**	**Glutathione S-transferase DHAR3**	2/0	[243]
*GSTU4*	AT2G29460	Glutathione S-transferase U4		[241,244]
*UGT74F2*	AT2G43820	UDP-glycosyltransferase 74F2		[244,246]
***UGT73C6***	**AT2G36790**	**UDP-glycosyltransferase 73C6**	0/1	[241,245]
*UGT74E2*	AT1G05680	UDP-glycosyltransferase 74E2		[241,245]
*UGT73B5*	AT2G15480	UDP-glycosyltransferase 73B5		[241,244]
*UGT75B1*	AT1G05560	UDP-glycosyltransferase 75B1		[241,244]
*CYP81F2*	AT5G57220	Cytochrome P450 81F2		[241]
***CYP87A2***	**AT1G12740**	**Photosynthetic NDH subunit of lumenal location 5**	1/1	[240]
*GSTF7*	AT1G02920	Glutathione S-transferase F7		[243]
*GSTF6*	AT1G02930	Glutathione S-transferase F6		[243,246]
*ABCB15*	AT3G28345	ABC transporter B family member 15		[242]
